# Risk factors for multidrug resistant bacteria and optimization of empirical antibiotic therapy in postoperative peritonitis

**DOI:** 10.1186/cc8877

**Published:** 2010-02-15

**Authors:** Pascal Augustin, Nathalie Kermarrec, Claudette Muller-Serieys, Sigismond Lasocki, Denis Chosidow, Jean-Pierre Marmuse, Nadia Valin, Jean-Marie Desmonts, Philippe Montravers

**Affiliations:** 1 Department of Anesthesiology and Surgical Intensive Care Unit, Hôpital Bichat-Claude Bernard, Université Paris VII Denis Diderot, Assistance Publique Hôpitaux de Paris, 46 rue Henri Huchard, 75877 Paris Cedex 18, France; 2 Department of Microbiology, Hôpital Bichat-Claude Bernard, Université Paris VII Denis Diderot, Assistance Publique Hôpitaux de Paris, 46 rue Henri Huchard, 75877 Paris Cedex 18, France; 3 Department of General Surgery, Hôpital Bichat-Claude Bernard, Université Paris VII Denis Diderot, Assistance Publique Hôpitaux de Paris, 46 rue Henri Huchard, 75877 Paris Cedex 18, France; 4 Department of Infectious Diseases, Hôpital Saint-Antoine, Université Paris VI, Assistance Publique Hôpitaux de Paris, 184 rue du Faubourg Saint-Antoine, 75571 Paris Cedex 12, France

## Abstract

**Introduction:**

The main objective was to determine risk factors for presence of multidrug resistant bacteria (MDR) in postoperative peritonitis (PP) and optimal empirical antibiotic therapy (EA) among options proposed by Infectious Disease Society of America and the Surgical Infection Society guidelines.

**Methods:**

One hundred patients hospitalised in the intensive care unit (ICU) for PP were reviewed. Clinical and microbiologic data, EA and its adequacy were analysed. The *in vitro *activities of 9 antibiotics in relation to the cultured bacteria were assessed to propose the most adequate EA among 17 regimens in the largest number of cases.

**Results:**

A total of 269 bacteria was cultured in 100 patients including 41 episodes with MDR. According to logistic regression analysis, the use of broad-spectrum antibiotic between initial intervention and reoperation was the only significant risk factor for emergence of MDR bacteria (odds ratio (OR) = 5.1; 95% confidence interval (CI) = 1.7 - 15; *P *= 0.0031). Antibiotics providing the best activity rate were imipenem/cilastatin (68%) and piperacillin/tazobactam (53%). The best adequacy for EA was obtained by combinations of imipenem/cilastatin or piperacillin/tazobactam, amikacin and a glycopeptide, with values reaching 99% and 94%, respectively. Imipenem/cilastin was the only single-drug regimen providing an adequacy superior to 80% in the absence of broad spectrum antibiotic between initial surgery and reoperation.

**Conclusions:**

Interval antibiotic therapy is associated with the presence of MDR bacteria. Not all regimens proposed by Infectious Disease Society of America and the Surgical Infection Society guidelines for PP can provide an acceptable rate of adequacy. Monotherapy with imipenem/cilastin is suitable for EA only in absence of this risk factor for MDR. For other patients, only antibiotic combinations may achieve high adequacy rates.

## Introduction

Postoperative peritonitis (PP) is a life-threatening complication of abdominal surgery with high rates of organ failure and mortality [1]. Adequate management of patients with PP requires supportive therapy of organ dysfunction, source control of infection with surgery and/or drainage, and antimicrobial therapy [2-5]. Because early and adequate antimicrobial therapy is an important goal in these high-risk patients [6,7], it is essential to take into account factors that modulate bacterial ecology and the susceptibility of causative organisms to ensure optimal management. Increased proportions of multidrug resistant (MDR) bacteria have been reported in this setting [1,8,9] and the role of previous antibiotic therapy in the emergence of these bacteria has been stressed [1,9]. Interestingly, few studies have addressed the therapeutic issues and difficulties related to the choice of empirical antibiotic therapy (EA) raised by these MDR microorganisms.

Based on these concerns, the aim of this study was first to identify risk factors for the presence of MDR bacteria in PP, and then to analyse the *in vitro *activities of some antimicrobial regimens proposed by guidelines from the Infectious Disease Society of America (IDSA) [2] and the Surgical Infection Society (SIS) [3] in order to propose antibiotic regimens providing adequate EA in the largest number of cases according to the identified risk factors of MDR bacteria.

## Materials and methods

### Study population

From January 2001 to December 2004, all consecutive adult patients with a diagnosis of PP requiring admission to a surgical intensive care unit (ICU) were prospectively included in a database, and their medical charts were retrospectively reviewed. PP was defined as a peritoneal infection occurring after an initial abdominal surgery (S0), and confirmed by macroscopic findings and positive bacterial fluid culture yielding at least one microorganism (bacteria or yeast) at reoperation. In patients who required multiple reoperations, only the first one was considered. All types of abdominal surgery were included except cases of complicated acute pancreatitis. Patients with PP with pure fungal infection were not analysed. According to French law, because this observational study did not modify the physicians' laboratory or clinical practices, no informed consent was required. The Institutional Review Board of Paris North Hospitals, Paris 7 University, AP-HP, reviewed and approved the study.

### Susceptibility testing and empirical antimicrobial therapy

Peritoneal fluid samples were systematically collected during reoperation and immediately sent to the bacteriology laboratory. Gram staining for direct examination and cultures were performed with identification and susceptibility testing for Gram-positive and Gram-negative bacteria. Antibiotic susceptibility was determined by the disk-diffusion method, according to the criteria of the Antibiogram Committee of the French Society for Microbiology [10]. *In vitro *susceptibility of nine antibiotics (amoxicillin/clavulanic acid (amox/clav); piperacillin/tazobactam (pip/taz); ceftazidime; imipenem/cilastatin; ciprofloxacin; gentamicin; amikacin and specifically metronidazole and vancomycin (for anaerobes and Gram-positive cocci)) was recorded for all bacteria. Results were expressed as proportions of susceptible bacteria for each antibiotic. Parenteral EA was systematically started at the time of reoperation according to the recommendations of our institutional protocol for PP. This protocol is based on treatment with a broad-spectrum beta-lactamin pip/taz or imipenem. Imipenem is selected for patients with severe peritonitis and/or previous antimicrobial therapy. The use of amikacin for spectrum broadening and synergistic combination is optional. The adjunction of vancomycin is considered in cases of prolonged hospital stay or methicillin-resistant staphylococcus or amoxicillin-resistant enterococcus carriage. Adequacy of EA was assessed according to the regimen used and the number of antibiotics in the case of combination therapy. Empirical antimicrobial therapy was considered adequate if, according to the susceptibility testing, all bacteria isolated were susceptible to at least one of the drugs administered. The antibiotic selection was considered to be adequate or inadequate strictly on the basis of the culture results obtained and did not reflect the authors' subjective assessment of appropriateness of care.

### Optimization of empirical antibiotic therapy

Analysis of antibiotic regimens classified as monotherapy or combination therapy (two-, three- and four-drug regimens) allowed the assessment of 17 potential regimens in order to determine suitable treatments providing adequate EA in the largest number of cases. This analysis was performed according to the presence or absence of MDR bacteria, and then according to the presence or absence of a risk factor for MDR strains found in our analysis. As the purpose of this study was to focus on antimicrobial therapy, fungi were not included in the definition of adequacy.

### Definitions

MDR bacteria were defined as: methicillin-resistant *Staphylococcus aureus *and coagulase-negative staphylococci (CNS); *Enterobacteriaceae *producing an extended-spectrum beta-lactamase or producing a cephalosporinase: and non-fermenting Gram-negative aerobes resistant to pip/taz, ceftazidime, or imipenem/cilastatin, or producing an extended-spectrum beta-lactamase (*Pseudomonas aeruginosa *and *Acinetobacter baumanii)*. In line with the IDSA and SIS guidelines considering broad-spectrum agents active against *P. aeruginosa*, and methicillin-susceptible and amoxicillin-susceptible *Enterococcus*, we arbitrarily defined pip/taz, imipenem/cilastatin, and fluoroquinolones as broad-spectrum antibiotics. Interval antibiotics (IA) were defined as antimicrobial agents administered between S0 and reoperation, at during at least 24 hours and started at least 24 hours before reoperation. The use of all-types of IA and broad-spectrum IA during this period was recorded in every case and constituted new variables for the analysis. The reason for their prescription was recorded.

### Data collected

The patients' medical charts were reviewed and the following information was collected: age; gender; severity of the underlying medical condition [11]; presence of chronic diseases (such as malignancy; diabetes mellitus; steroid or immunosuppressive therapy for inflammatory bowel disease); previous hospitalization or antibiotic therapy within three months before S0; characteristics of S0, if performed in another institution, its type, route and wound class [12]; and use of IA. Parameters collected within the first 48 hours after ICU admission were: temperature; acute physiology and chronic health evaluation (APACHE) II score [13]; Sequential Organ Failure Assessment (SOFA) score [14]; organ failures assessed following Knauss definitions [15]; etiology and primary site (above or below transverse mesocolon) of the infection responsible for PP and time to reoperation; identification of pathogens in peritoneal fluid; and results of antimicrobial susceptibility tests.

### Outcome

Patient outcome was recorded as the number of reoperations, duration of mechanical ventilation, ICU length of stay, and ICU mortality. The prognosis was assessed by taking into account the presence of MDR organisms and the adequacy of EA.

### Statistical analysis

Results are expressed as mean ± standard deviation, and as percentages for categorical variables. All analyses were performed using the Statview software package (version 5.0; SAS institute Inc, Cary, NC, USA). As the primary objective of the study was to determine risk factors and outcome of PP patients with MDR bacteria, the group of patients with MDR bacteria (called MDR group) was compared with the group of patients with 'other' bacteria (called other group). Secondly, the impact of broad-spectrum IA on susceptibility of microorganisms collected from peritoneal samples was analyzed. Univariate analysis was performed using Student's *t*-test or Wilcoxon's rank sum test, as appropriate for continuous variables, and the Chi squared or Fisher's exact test, as appropriate, for categorical variables. All variables with a *P *value less than 0.10 in the univariate analysis were entered into a multivariate logistic regression analysis. Odds ratio (OR) and 95% confidence intervals (CI) were calculated. Statistical significance was defined as *P *< 0.05.

## Results

### Demographics on admission to ICU

During the study period, 107 patients with PP were admitted to our ICU. Seven patients were excluded because only fungi were found on culture. Epidemiologic characteristics, clinical status of the 100 patients on admission and clinical findings at the time of reoperation are shown in Tables 1 and 2. Initial surgery was digestive in 80 cases, hepatobiliary in 5 cases, urologic in 7, mixed urologic/digestive in 3 cases, and gynaecologic in 8 cases. In this study population, the presence of MDR bacteria was reported in 41 PP patients and 59 PP patients were free of MDR strains. According to univariate analysis, factors associated with the presence of MDR bacteria in peritoneal samples at the time of PP were emergent initial surgery, contaminated or infected initial surgery, prior antibiotic therapy before S0, IA and broad-spectrum IA. When these variables were entered into a logistic regression model, the use of broad-spectrum IA was the only significant risk factor for emergence of MDR bacteria (OR = 5.1; 95% CI = 1.7 to 15; *P *= 0.0031).

**Table 1:** Demographic characteristics at initial surgery S0, and interval antibiotic therapy in the 100 patients with PP.

Variable	Patients with MDR bacteria (n = 41)	Patients with other bacteria (n = 59)	*P*
Age (year), mean ± SD	63 ± 17	60 ± 16	0.37
Gender, male, n (%)	24 (59)	32 (54)	0.83
Severity of underlying disease			
Fatal (within 5 years), n (%)	16 (39)	21 (36)	0.92
Malignancy, n (%)	17 (41)	22 (37)	0.86
Diabetes mellitus, n (%)	7 (17)	11 (19)	0.91
Steroids or immunosuppressive therapy, n (%)	15 (37)	27 (46)	0.36
Initial surgery			
In emergency, n (%)	20 (49)	15 (25)	0.01
Contaminated or infected wound class, n (%)	25 (61)	19 (32)	0.016
Prior hospitalization (within 3 months prior S0), n (%)	24 (59)	29 (49)	0.24
Prior antibiotic therapy (within 3 months, prior S0), n (%)	18 (44)	13 (22)	0.02
Interval antibiotics, n (%)	33 (80)	35 (59)	0.026
Broad-spectrum interval antibiotics, n (%)	25 (61)	11 (19)	0.0001

MDR, multidrug resistant; PP, postoperative peritonitis; SD, standard deviation; S0, initial abdominal surgery.

**Table 2:** Characteristics and clinical findings at reoperation in the 100 patients with PP

Variable	Patients with MDR bacteria (n = 41)	Patients with other bacteria (n = 59)	*P*
APACHE II, mean ± SD	21 ± 7	21 ± 7	0.90
SOFA, mean ± SD	7 ± 4	7 ± 4	0.63
≥ 1 organ failures, n (%)	32 (78)	48 (81)	0.68
Vasopressor support, n (%)	26 (63)	41 (69)	0.38
Time to reoperation (days), mean ± SD	13 ± 16	10 ± 11	0.55
Mechanisms of PP			
Anastomotic leakage, n (%)	14 (34)	20 (34)	0.98
Perforation, n (%)	9 (22)	21 (36)	0.14
Miscellaneous, n (%)	8 (20)	10 (17)	0.98
Unknown cause, n (%)	10 (24)	8 (14)	0.27
Source of PP			
Lower intestinal tract, n (%)	16 (39)	32 (54)	0.67

APACHE, acute physiology and chronic health evaluation; MDR, multidrug resistant; PP, postoperative peritonitis; SD, standard deviation; SOFA, Sequential Organ Failure Assessment.

### Susceptibility testing and interval antimicrobial therapy

A total of 269 bacteria were cultured from peritoneal fluid (Table 3). Twenty five yeasts were isolated including *Candida albicans *(n = 12), *Candida glabrata *(n = 7) and *Candida tropicalis *(n = 4). Most patients (n = 68) received all-types of IA, and 35 of them received broad-spectrum IA. The main reasons for IA were contaminated or septic initial surgery, suspicion or occurrence of PP (n = 26), and new focus of infection (n = 21) including 12 cases of pneumonia. The distribution of bacteria according to the use of broad-spectrum IA therapy is presented in Table 4. The number of bacteria cultured from peritoneal fluid, was not different when broad-spectrum IA therapy had been administered (2.5 ± 1.7 *vs *2.8 ± 2.1, *P *= 0.22). In these patients, we observed that cultures of peritoneal fluid samples exhibited a trend toward increased proportions of monomicrobial samples (20% *vs *8% in patients without broad spectrum IA therapy, *P *= 0.18), with a higher number of MDR microorganisms, mainly due to resistant *Enterobacteriaceae *and methicillin-resistant CNS (*P *< 0.05 for both cases). All-types of IA were associated with a decreased number of bacteria (2.4 ± 1.5 *vs *3.4 ± 2.4, *P *= 0.001) and PP was more often monomicrobial PP (28% *vs *3%, *P *= 0.001).

**Table 3:** Bacteria isolated from peritoneal fluid in 100 episodes of postoperative peritonitis

Microorganisms	Number of strains n (%)	Monomicrobial infection
**Gram-positive bacteria**	**108 (40)**	
Enterococci	50 (19)	
*E. faecium*	11 (4)	
Other	39 (14)	1
Streptococci	30 (11)	
Staphylococci	28 (10)	
*S. aureus*	7 (3)	
Coagulase-negative staphylococci	21 (8)	3
**Gram-negative bacteria**	**119 (44)**	
Enterobacteriaceae	101 (37)	
*Escherichia coli*	49 (18)	4
*Enterobacter *species	22 (8)	1
*Klebsiella *species	13 (5)	
*Morganella morganii*	7 (3)	1
*Proteus *species	5 (2)	
*Citrobacter *species	5 (2)	
*Pseudomonas aeruginosa*	16 (6)	1
*Acinetobacter baumannii*	2 (1)	
***Miscellaneous***	**6 (2)**	**1**
***Anaerobes***	**36 (13)**	
*Bacteroides *species	20 (7)	

Total bacteria	269 (100)	12

**Table 4:** Numbers and percentages of bacteria responsible for PP according to the use of broad-spectrum IA

Microorganisms	Patients without broad-spectrum IA (n = 65)	Patients with broad-spectrum IA (n = 35)
**Multidrug resistant bacteria, n(%)**	**24 (13)**	41 (48) *
*Enterobacteriaceae, n(%)*	9 (5)	16 (19) *
*Pseudomonas aeruginosa, n(%)*	3 (2)	5 (6)
*Acinetobacter baumannii, n(%)*	1 (1)	1 (1)
Enterococci, n(%)	4 (2)	3 (3)
Methicillin-resistant *S. aureus, n(%)*	4 (2)	3 (3)
Methicillin-resistant CNS, n(%)	3 (2)	13 (15) *
**Other bacteria, n(%)**	**160 (87)**	**44 (52)**
*Enterobacteriaceae, n(%)*	69 (37)	11 (13) *
*Pseudomonas aeruginosa, n(%)*	2 (1)	6 (7)
Enterococci, n(%)	31 (17)	12 (14)
Streptococci, n(%)	27 (15)	5 (6)
Staphylococci, n(%)	5 (3)	0
Other pathogens, n(%)	26 (14)	10 (12)
**Total number of bacteria**	**184 (100)**	85 (100) *

CNS, coagulase negative staphylococci; IA, interval antibiotic therapy; PP, postoperative peritonitis; * *P *< 0.05 *vs *group without broad-spectrum IA.

Proportions of susceptible Gram-negative and Gram-positive strains have been evaluated. Among the various antibiotics tested, imipenem/cilastatin and amikacin were the most consistently active against aerobic Gram-negative bacteria in all patients, whereas the efficacy of pip/taz (87% *vs *40%, *P *< 0.0001) and ceftazidime (87% *vs *60%, *P *= 0.009) was markedly reduced in patients with broad-spectrum IA therapy. Vancomycin was the agent most frequently active against Gram-positive bacteria in all patients, except in one case of a naturally resistant *Enterococcus casseliflavus *strain. Following broad-spectrum IA therapy, staphylococci were resistant to beta-lactams and ciprofloxacin. The 36 cultured anaerobes had susceptibility rates of 87%, 93%, 93% and 100% toward amox/clav, pip/taz, metronidazole, and imipenem/cilastatin, respectively. Among the 20 *Bacteroides *strains, four were resistant to amox/clav, two to pip/taz and one to metronidazole.

### Empirical antimicrobial therapy

We analysed EA prescribed at the time of reoperation in the 100 PP patients: monotherapy in 53 cases (45 pip/taz; 5 imipenem), double-drug combinations in 32 cases (13 based on pip/taz; 10 based on imipenem), and triple-drug combinations in 13 cases (4 based on pip/taz; 4 based on imipenem). Adequacy rates were 64%, 66%, and 62%, for monotherapies, double-drug combinations, and triple-drug combinations, respectively.

Pip/taz (n = 66) and imipenem/cilastatin (n = 23) were the main agents prescribed. Imipenem/cilastatin was more frequently administered than pip/taz in seriously ill patients (SOFA score 6 ± 4 *vs *9 ± 3, *P *= 0.005), and in the case of prior broad-spectrum IA therapy between S0 and reoperation (87% for imipenem *vs *65% for pip/taz; *P *= 0.04). A higher SOFA score was also associated with prescriptions of combinations rather than monotherapy (6 ± 4 for monotherapy *vs *8 ± 4 for combination; p = 0.03). Three allergic patients received triple-drug combinations without beta-lactams. One patient with previous colonization by a multiresistant strain of *P. aeruginosa *received a four-drug combination (imipenem/cilastatin + vancomycin + aminoglycosides + colistin). One patient received antifungal therapy only because of previous fungal colonization and negative direct examination of peritoneal fluid.

Adequate EA was achieved in 64% of cases. Adequacy of EA decreased significantly in patients with MDR bacteria, as compared with patients with 'other bacteria' (39% *vs *81%, *P *< 0.0001).

### Optimization of empirical antibiotic therapy

**Figure 1:**
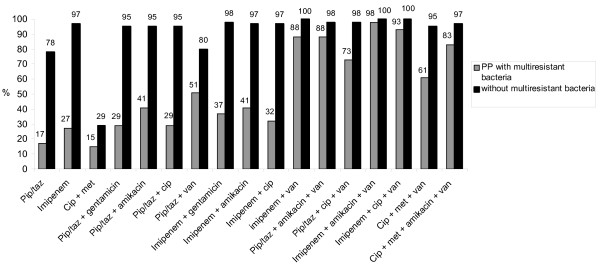
**Adequacy rates of 17 theoretical antibiotic regimens according to the presence or absence of multidrug resistant bacteria**. cip, ciprofloxacin; met, metronidazole; pip/taz, piperacillin/tazobactam; PP, postoperative peritonitis.

**Figure 2:**
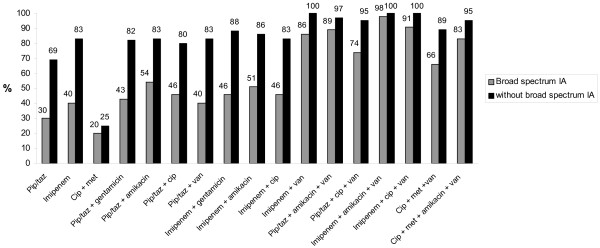
**Adequacy rates of 17 theoretical antibiotic regimens according to the presence or absence of broad-spectrum IA**. cip, ciprofloxacin; met, metronidazole; IA, interval antibiotics; pip/taz, piperacillin/tazobactam.

Evaluation of the adequacy rates of 17 theoretical regimens in the 100 episodes of PP according to the presence or absence of MDR bacteria, and according to the prescription of a broad-spectrum IA are shown in Figures 1 and 2, respectively. Only combination regimens including vancomycin achieved empirical therapy adequacy rates higher than 80%. Regimens based on imipenem/cilastatin obtained the highest adequacy rate. In patients with broad-spectrum IA, monotherapy with imipenem/cilastatin provided only poor adequacy rates, but was suitable for patients without broad-spectrum IA. Monotherapy with pip/taz gave poor results even in patients without broad-spectrum IA.

### Outcome

Forty-four patients had a reoperation after R1 (first repoperation at ICU admission) because of persistent peritonitis. ICU mortality rate was 31%. Mortality did not differ between patients with adequate EA and others (30% *vs *31%, *P *= 0.9), and between patients with PP caused by MDR bacteria and other bacteria (29% for MDR group *vs *35% for others, *P *= 0.69). The mean duration of antibiotic therapy (10 ± 4 days *vs *12 ± 6 days, *P *= 0.07), mechanical ventilation (10 ± 9 days *vs *11 ± 16 days, *P *= 0.6), length of ICU stay (16 ± 11 days *vs *20 ± 19 days, *P *= 0.2), as well as the number of reoperations (0.8 ± 1.4 *vs *0.8 ± 1, *P *= 0.9) were similar in patients with adequate EA and other patients, respectively. No outcome difference was observed between patients with MDR bacteria and patients with other microorganisms.

## Discussion

In this single-center study, broad-spectrum IA prescribed between initial surgery and reoperation for PP was associated with the emergence of MDR bacteria in peritoneal samples, mostly *Enterobacteriaceae *and CNS. Only combination EA adequately targeted all bacteria.

Guidelines for antibiotic therapy for severe intra-abdominal infections issued by the IDSA [2] and SIS [3] provide a list of regimens suitable for the treatment of peritonitis, but these recommendations do not specifically address the case of PP. These statements indicate that local nosocomial resistance patterns should guide EA.

The role of antibiotic therapy in the modification of bowel flora and in the selection of MDR bacteria is well known [16,17], but has been rarely assessed in PP [1,9]. In this setting, IA use reported in 62 to 80% of PP patients [1,8,9] could play an important role in the selection of MDR strains. To our knowledge, a significant link between broad-spectrum IA and emergence of MDR *Enterobacteriaceae *and CNS has not been previously described in patients with PP [1,8,9].

The bacteriologic profiles found in our population are similar to those previously described in PP [1,8,9,18-20]. Interestingly, the proportions of MDR organisms in our institution appear to have remained fairly stable over the past 10 years [8] and are situated in the same range as those observed in another French institution [9]. The proportion of enterococci is situated within the usual range in our population [1,8,9,18] without vancomycin-resistant strains [9,20]. A high prevalence of CNS was observed, as in previous reports [1,8,9,18,21,22]. The majority of studies on PP did not identify the type of staphylococci (CNS or *S. aureus*). We may hypothesise that some authors do not record CNS as a pathogen. Current knowledge does not allow differentiation of microorganisms with a clinical relevance from suspected 'non-pathogenic' strains. Enterococci and CNS share a number of similarities, such as presence at low concentration in peritoneal fluid, low pathogenicity and presence as commensals in the bowel flora. They are also considered to be typical representatives of tertiary peritonitis in association with *Pseudomonas *and *Candida *[3,4]. Although there is a general agreement to target enterococci in PP antibiotic therapy, there is no therapeutic statement regarding CNS [2,3]. We deliberately chose to target these microorganisms in the EA of PP patients. This somewhat crude attitude therefore corresponds to the lowest common denominator for clinicians with the assurance of targeting all pathogenic strains.

Recent guidelines emphasize the importance of early EA targeting all microorganisms followed by rapid de-escalation after microbiologic identification of pathogens and susceptibility testing [2,3,6,7]. In line with IDSA and SIS guidelines [2,3], our local recommendations for EA were mainly based on a broad-spectrum monotherapy. In our population, not all regimens proposed for EA are suitable for all patients. Furthermore, our data suggest that none of the monotherapies proposed would provide a high rate of adequacy [2-4]. Consequently, we assume that patients with risk factors for MDR strains should receive antibiotic combinations, whereas broad-spectrum monotherapy should be restricted to those without broad-spectrum IA. Interestingly, the spectrum of activity of pip/taz does not seem to be sufficient even in the subgroup of patients with no risk factors for MDR bacteria. This result is not consistent with a multicenter trial that reported similar results for pip/taz alone or combined with aminoglycosides [19]. However, this study was performed 10 years ago and may no longer reflect current concerns [20,23]. Our results suggest that routine identification and susceptibility testing of peritoneal samples remain mandatory for subsequent de-escalation antibiotic therapy, to report prevalence of resistance and to detect trends over time.

Inadequate antimicrobial therapy has been shown to prolong hospitalisation and is associated with increased clinical failures and higher mortality rates [7,8,24,25]. This link between inadequate EA and outcome was not observed in this study, as in several other recent studies of nosocomial peritoneal infections [1,9,18,20,26]. This apparent contradiction could be attributed to the definition of inadequacy, which takes into account all of the strains isolated, including enterococci or CNS whose pathogenicity remains a subject of debate. We may also hypothesise that our previous results were wrong or obtained by chance [8]. A more plausible explanation could be the changing trends in patients' characteristics, improvement of surgical techniques and intensive care management over the years. The weight of antibiotic therapy in patient outcome may have decreased. Indeed, the more important part of management of peritonitis remains surgery to control the source of infection and decrease bacterial load. Despite the uncertain links between prognosis and inadequacy of EA, we assume that an EA targeting all pathogens is a reasonable goal to be achieved in line with current recommendations [6,7]. However, the benefits of broad-spectrum combinations must be balanced with their potential drawbacks, such as emergence of resistance, high costs, and toxic effects.

This study may present a number of limitations. Even if our results are similar to observations reported at the same period in two prospective French studies, a single-center study [9] and a susceptibility survey performed in 25 French institutions [20], our results obtained in a single-center study cannot provide any definitive conclusions for other institutions. This point is of particular value for other countries. In fact, very few studies have been conducted outside of France and reported approximately the same bacteriologic profiles [1], but data on susceptibility patterns are scarce and weak [27]. However, this study emphasizes the need to evaluate bacteriologic profiles in each institution. The definition of adequacy is based purely on microbiologic criteria and *a priori *assumptions and does not take yeasts into account. Agents other than those reported here could have been chosen, but these drugs were not routinely used and were not systematically tested in our microbiology laboratory.

## Conclusions

Our data suggest that identification of risk factors for MDR strains could help to improve the adequacy of early EA in PP patients. In our population, patients receiving IA therapy seem to be at risk of emergence of MDR strains and at high risk of inadequate EA. In presence of this risk factor, only combination therapies provided a high probability of adequate EA. Such a policy of optimisation of EA should be discussed locally based on analysis of resistance patterns of PP, so as to identify among options proposed by guidelines, regimens providing acceptable adequacy rates. Longitudinal evaluation is also necessary to follow the evolution of resistance patterns.

## Key messages

• The high rate of MDR bacteria in PP is confirmed.

• Broad-spectrum IA between initial surgery and reoperation for PP is a risk factor for emergence of MDR bacteria.

• Not all antibiotic regimens proposed by IDSA or SIS for PP can provide high rate of adequacy.

• Pip/taz alone may be inadequate in a large number of cases even in absence of the risk factor for MDR.

• In presence of the risk factor for MDR, only combination regimens can provide high rate of adequacy.

## Abbreviations

amox/clav: amoxicillin/clavulanic acid; APACHE: acute physiology and chronic health evaluation; CI: confidence intervals; CNS: coagulase-negative staphylococci; EA: empirical antibiotic therapy; IA: interval antibiotics; ICU: intensive care unit; IDSA: Infectious Disease Society of America; MDR: multidrug resistant; OR: odd ratio; pip/taz: piperacillin/tazobactam; PP: postoperative peritonitis; S0: initial abdominal surgery; SIS: Surgical Infection Society; SOFA: Sequential Organ Failure Assessment.

## Competing interests

The authors declare that they have no competing interests.

## Authors' contributions

PA drafted the manuscript and helped in the data collection. NK drafted the manuscript, helped in the data collection, and in the study conception. CMS had a contribution for bacteriologic data and manuscript revision. SL had a contribution in the manuscript preparation and data collection. DC had a contribution in the manuscript preparation and data collection. JPM had a contribution in the manuscript preparation and data collection. NV contributed in the manuscript and statistical revision. JMD has been involved in the conception of the study. PM conceived the design and coordination and helped to draft the manuscript. All authors read and approved the final manuscript.
